# Aberrant brain functional network strength related to cognitive impairment in age-related hearing loss

**DOI:** 10.3389/fneur.2022.1071237

**Published:** 2022-12-21

**Authors:** Shaoyun Zhu, Jiajie Song, Wenqing Xia, Yuan Xue

**Affiliations:** ^1^Department of Ultrasound, Nanjing Pukou Central Hospital, Pukou Branch Hospital of Jiangsu Province Hospital, Nanjing, China; ^2^Department of Radiology, Nanjing Pukou Central Hospital, Pukou Branch Hospital of Jiangsu Province Hospital, Nanjing, China; ^3^Department of Endocrinology, Nanjing First Hospital, Nanjing Medical University, Nanjing, China; ^4^Department of Otolaryngology, Nanjing Pukou Central Hospital, Pukou Branch Hospital of Jiangsu Province Hospital, Nanjing, China

**Keywords:** age-related hearing loss, brain network, degree centrality, functional network strength, cognitive impairment

## Abstract

**Purpose:**

Age-related hearing loss (ARHL) is a major public issue that affects elderly adults. However, the neural substrates for the cognitive deficits in patients with ARHL need to be elucidated. This study aimed to explore the brain regions that show aberrant brain functional network strength related to cognitive impairment in patients with ARHL.

**Methods:**

A total of 27 patients with ARHL and 23 well-matched healthy controls were recruited for the present study. Each subject underwent pure-tone audiometry (PTA), MRI scanning, and cognition evaluation. We analyzed the functional network strength by using degree centrality (DC) characteristics and tried to recognize key nodes that contribute significantly. Subsequent functional connectivity (FC) was analyzed using significant DC nodes as seeds.

**Results:**

Compared with controls, patients with ARHL showed a deceased DC in the bilateral supramarginal gyrus (SMG). In addition, patients with ARHL showed enhanced DC in the left fusiform gyrus (FG) and right parahippocampal gyrus (PHG). Then, the bilateral SMGs were used as seeds for FC analysis. With the seed set at the left SMG, patients with ARHL showed decreased connectivity with the right superior temporal gyrus (STG). Moreover, the right SMG showed reduced connectivity with the right middle temporal gyrus (MTG) and increased connection with the left middle frontal gyrus (MFG) in patients with ARHL. The reduced DC in the left and right SMGs showed significant negative correlations with poorer TMT-B scores (r = −0.596, *p* = 0.002; r = −0.503, *p* = 0.012, respectively).

**Conclusion:**

These findings enriched our understanding of the neural mechanisms underlying cognitive impairment associated with ARHL and may serve as a potential brain network biomarker for investigating and predicting cognitive difficulties.

## 1. Introduction

Age-related hearing loss (ARHL) is a major public issue that affects elderly adults (1). There is growing evidence to suggest that hearing deprivation can precede the onset of dementia by 5 to 10 years since it often leads to social isolation, depression, anxiety, and communication problems (2). A 12-year follow-up study indicated that the risk of dementia associated with hearing loss (ages ranging from 36 to 90) was 36.4% (3). However, the neural substrates underlying cognitive impairment with ARHL need to be elucidated.

Previous neuroimaging studies have proven that ARHL shows a constellation of changes in the auditory and non-auditory cortices. It is increasingly recognized that ARHL is related to structural and functional changes in the central auditory pathway and other areas of the central nervous system (4). In terms of the global brain, cortical thinning and reduced gray matter volume have been found in ARHL subjects (5, 6). Ren et al. also found that abnormal spontaneous neural activity was frequency dependent and correlated with cognition by exploring all frequency bands of oscillation (7). Some studies have shown that the functional reorganization of the auditory cortex (8), cingulo-opercular network (9), motor (10), visual (11), and attention networks (12) in ARHL could be responsible for cognitive and neural functioning and, in turn, affect auditory processing.

Degree centrality (DC) is a voxel-wise data-driven method that can quantify the importance of each node in a brain network. This graph theory-based network analysis can assess the network centrality without *a priori* selection of nodes or networks of interest (13). It has been recently used to assess the pathophysiological mechanism of various neurological and psychiatric diseases (14, 15). DC has more advantages than other algorithms, such as seed-based functional connectivity (FC) and independent component analysis (ICA), which depend on specific components of interest for connectivity patterns. Thus, to unravel the details of the brain functional network strength in ARHL, we sought to evaluate the brain regions that show aberrant functional connectivity across all brain networks in patients with ARHL using the DC method. Our study further analyzed the DC characteristics related to cognitive impairment in ARHL and tried to recognize the key nodes that contribute significantly. Subsequent FC analysis was analyzed using significant DC nodes as seeds to detect their relationships with other brain regions. We hypothesized that the intrinsic dysconnectivity patterns of cognition-specific brain regions might play a pivotal role in the brain functional network strength of patients with ARHL. The DC and subsequent FC patterns could be disrupted in ARHL linked to cognitive impairment.

## 2. Materials and methods

### 2.1. Subjects

We recruited 27 patients with ARHL from the otolaryngology department of our hospital and 23 age-, sex-, and education-matched healthy controls from the local community *via* advertisements. A pure-tone audiometry (PTA) test was computed to evaluate the hearing threshold, and the diagnosis of ARHL was defined as a PTA value of >25 dB at high frequencies. The thresholds of both ears in healthy controls were <25 dB at six frequencies (0.25, 0.5, 1, 2, 4, and 8 kHz). The tympanometry test was conducted to confirm the function of the middle ear. Subjects were excluded from the present research if they (1) suffered from pulsatile tinnitus, Meniere's disease, conductive deafness, vertigo, Parkinson's disease, mild cognitive impairment (MCI), Alzheimer's disease, neurological disorders, and major illnesses; (2) had a history of brain injury, drug addiction, smoking, or alcoholic addition; or (3) had MRI contraindications, such as cochlear implants, pacemakers, or prosthetic valves. This prospective study was approved by the research ethics committee of Nanjing Medical University. All participants provided written informed consent before the experiment.

### 2.2. Cognitive tests

All participants underwent a detailed battery of standardized neuropsychological tests to reveal their cognitive status, mainly focusing on memory, attention, and executive functions, including the Mini-Mental State Exam (MMSE), Montreal Cognitive Assessment (MoCA), Auditory Verbal Learning Test (AVLT), Complex Figure Test (CFT), Digit Span Test (DST), Trail Making Test (TMT-A and B), Clock-Drawing Test (CDT), Digit Symbol Substitution Test (DSST), and Verbal Fluency Test (VFT). The Self-Rating Anxiety Scale (SAS) and Self-Rating Depression Scale (SDS) were used to assess mental conditions.

### 2.3. MRI acquisition

All MRI data were acquired using a 3.0 Tesla MRI scanner (MAGNETOM Vida, Siemens Healthcare, Erlangen, Germany) with a 64-channel phased-array head coil, including 3D-T1 and blood oxygen level-dependent (BOLD) sequences. Every subject was asked to lie quietly, keep their eyes closed, remain awake, and avoid thinking about anything special during the scanning procedure. The noise of MRI scanning was reduced using earplugs. BOLD was acquired using a gradient echo-planar imaging sequence with 240 time points as follows: repetition time (TR) = 2,000 ms, echo time (TE) = 30 ms, slices = 33, thickness = 4 mm, gap = 0 mm, the field of view (FOV) = 192 × 192 mm, acquisition matrix = 64 × 64, and flip angle (FA) = 90°. The scanning time for the BOLD sequence lasted 8 min and 8 s. The parameters of 3D-T1-weighted imaging were as follows: TR/TE = 5000/2.98 ms, slices = 176, thickness = 1 mm, gap = 0 mm, FA = 90°, acquisition matrix = 256 × 256, and FOV = 256 × 256 mm. The scanning time for the 3D-T1 sequence lasted 5 min and 29 s.

### 2.4. Data processing

Functional data were preprocessed using DPABI (http://rfmri.org/dpabi) and SPM 12 (http://www.fil.ion.ucl.ac.uk/spm) in MATLAB (R2013b). The preprocessing steps of functional data contain the following steps: (1) removing the first 10 time points to minimize the effect of signal instability; (2) slice timing; (3) realignment for head motion correction; (4) segmentation; (5) normalization to a standard template; (6) regressing six motion parameters, six temporal derivatives, and 12 corresponding squared items using the Friston-24 parameter; (7) smoothing with 8-mm full width at half-maximum Gaussian kernel; and (8) detrending and filtering (0.01–0.08). Subjects with a translational or rotational head motion of >2.0 mm or 2.0° in any direction were excluded. In this study, no subjects were excluded due to excessive head motion.

Then, we computed degree centrality (DC) analysis using DPABI, which is a graph theory-based technique. The BOLD time course of each voxel was extracted, and Pearson's correlation coefficient (r) between any pair of brain voxels was calculated. A matrix of Pearson's correlation coefficients was obtained to construct the whole-brain functional connectivity matrix for each participant at r > 0.25 (16). Finally, FC analysis was conducted using the significant DC nodes as seeds to reflect abnormalities in the core brain hub and its relationships with other brain areas.

### 2.5. Statistical analysis

The normality distribution of demographic characteristics and cognitive assessments was checked by using the Kolmogorov–Smirnov method. The intergroup difference in age was analyzed by using an independent-sample *t*-test. The comparison of sex between groups was conducted by a chi-square test. The Mann–Whitney *U*-test was applied for the comparisons between groups in years of education and cognitive performance. SPSS software (version 22.0, SPSS Inc., Chicago, IL, United States) was utilized for the statistical analyses. The significant *p*-value was set at <0.05.

A one-sample *t*-test was conducted to assess patterns of DC and FC maps using DPABI software. A two-sample *t*-test was used to assess different DC and FC maps between groups using the Gaussian random field (GFR) correction that was used to adjust for multiple comparisons using DPABI software (two-tailed, voxel-level: *p* < 0.01, GRF correction, cluster-level: *p* < 0.05). Finally, Pearson's correlation coefficients between abnormal DC and FC values and cognitive scores were analyzed using SPSS. Partial correlations were computed by adjusting for age, sex, and education level.

## 3. Results

### 3.1. Demographic and cognitive scores

**Figure 1:**
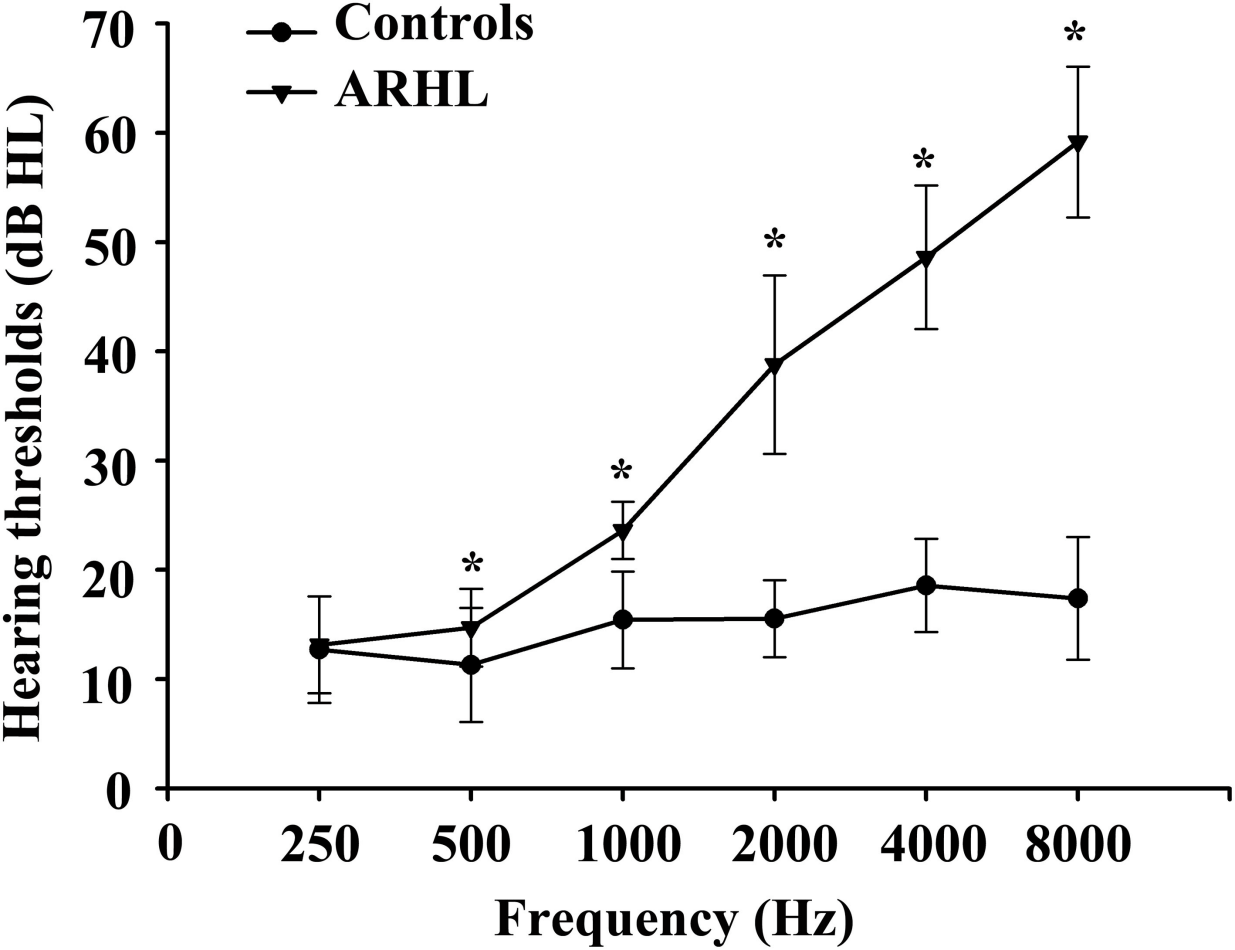
Hearing thresholds of ARHL and healthy controls using PTA test. Data are shown as mean ± SD. PTA, pure-tone audiometry. ^*^Means significant differences of hearing thresholds between two groups.

Detailed information about the demographic and neuropsychological data is shown in Table 1. There were no significant differences in terms of age, sex, or education. Compared with healthy controls, the hearing thresholds of the left and right ears in patients with ARHL were higher (*p* < 0.001), especially at high frequencies (Figure 1). In addition, patients with ARHL performed worse on the TMT-B, which primarily concentrated on attention and executive function. No significant differences in other cognitive scores were observed between patients with ARHL and healthy controls.

**Table 1:** Demographic and cognitive data in patients with ARHL and healthy controls.

	**ARHL patients (*n* = 27)**	**Healthy controls (*n* = 23)**	***p*-value**
Age (years)	64.11 ± 6.74	61.61 ± 3.54	0.116
Sex (Male: Female)	13/14	12/11	0.777
Education level (years)	10.15 ± 1.99	10.43 ± 1.44	0.569
PTA (Left, dB HL)	33.33 ± 3.89	15.07 ± 2.45	0.000^*^
PTA (Right, dB HL)	33.14 ± 6.55	15.25 ± 3.25	0.000^*^
PTA (Mean, dB HL)	33.23 ± 3.55	15.16 ± 1.91	0.000^*^
MMSE (scores)	28.93 ± 1.17	28.87 ± 1.39	0.877
MoCA (scores)	25.78 ± 1.80	26.48 ± 1.88	0.186
CDT (scores)	3.52 ± 0.58	3.48 ± 0.51	0.797
CFT (scores)	34.43 ± 1.64	34.54 ± 1.85	0.812
CFT-delay (scores)	16.67 ± 2.86	17.41 ± 4.34	0.470
TMT-A (seconds)	71.81 ± 21.44	65.96 ± 21.49	0.341
TMT-B (seconds)	235.52 ± 40.25	182.52 ± 54.95	0.000^*^
AVLT (scores)	34.81 ± 8.58	36.43 ± 5.34	0.437
VFT (scores)	13.90 ± 1.53	11.79 ± 1.78	0.296
DST (scores)	11.07 ± 1.58	11.78 ± 2.35	0.210
DSST (scores)	70.04 ± 9.12	68.30 ± 7.91	0.480
SDS (scores)	39.93 ± 10.24	39.09 ± 8.86	0.760
SAS (scores)	37.56 ± 6.25	35.35 ± 6.63	0.232

ARHL, age-related hearing loss; PTA, pure-tone audiometry; MMSE, Mini-Mental State Exam; MoCA, Montreal Cognitive Assessment; CDT, Clock-Drawing Test; CFT, Complex Figure Test; TMT, Trail Making Test; AVLT, Auditory Verbal Learning Test; VFT, Verbal Fluency Test; DST, Digit Span Test; DSST, Digit Symbol Substitution Test; SDS, Self-Rating Depression Scale; SAS, Self-Rating Anxiety Scale. ^*^ *p* < 0.001.

### 3.2. DC and FC analyses

**Figure 2:**
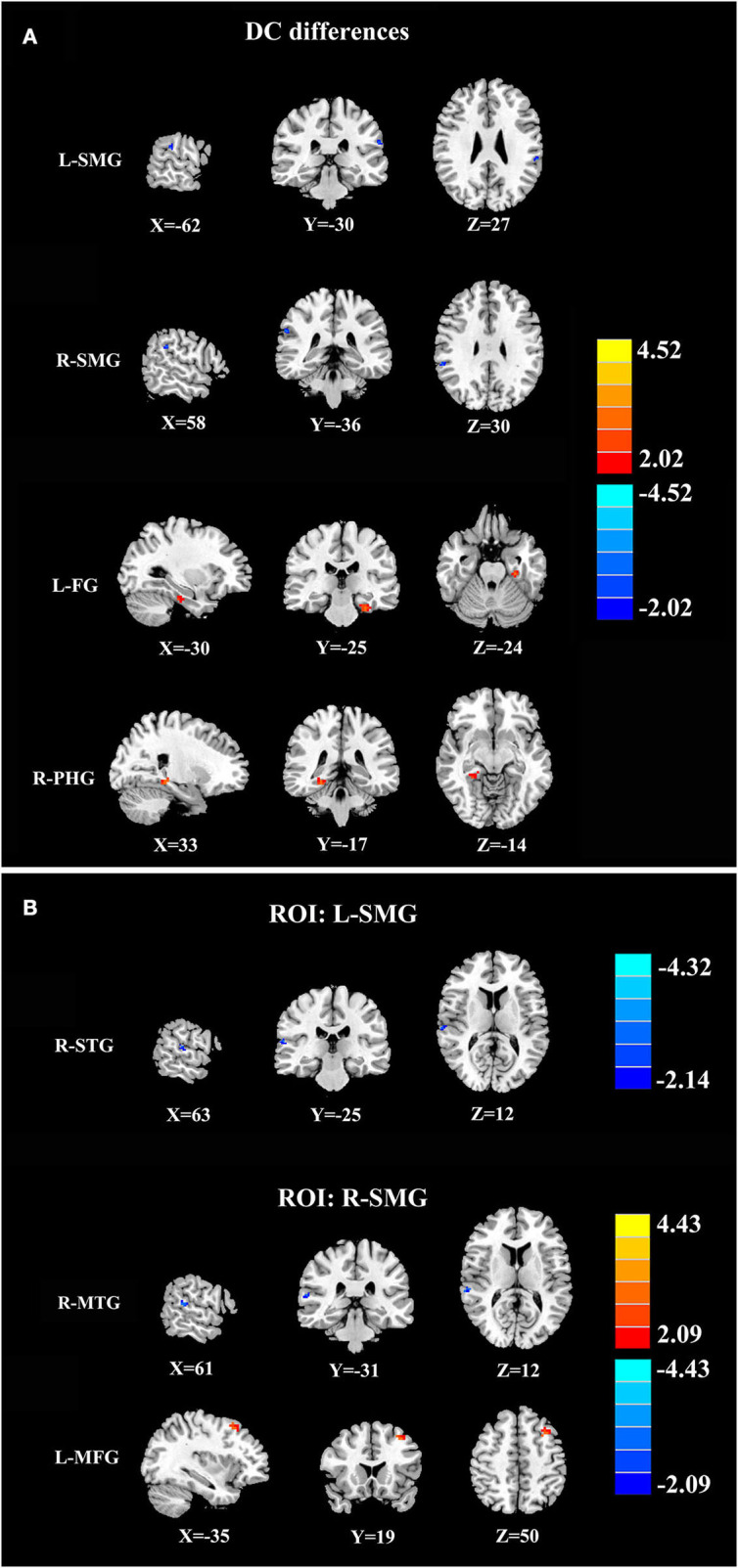
DC and FC results. **(A)** DC analysis between patients with ARHL and healthy controls. Compared with healthy controls, patients with ARHL exhibited reduced DC in the left and right supramarginal gyrus (L-SMG and R-SMG) as well as enhanced DC in left fusiform gurus (L-FG) and right parahippocampal gyrus (R-PHG). **(B)** FC analysis between patients with ARHL and healthy controls. With the seed set at L-SMG, patients with ARHL showed lower FC with the right superior temporal gyrus (R-STG). With the seed set at R-SMG, patients with ARHL showed reduced FC with the right middle temporal gyrus (R-MTG) and increased FC with the left middle frontal gyrus (L-MFG). The significant cluster-level *p* was set at <0.05 with Gaussian random field (GFR) correction.

Compared with healthy controls, patients with ARHL exhibited reduced DC in the bilateral supramarginal gyrus (SMG). Moreover, patients with ARHL showed enhanced DC in the left fusiform gyrus (FG) and right parahippocampal gyrus (PHG). According to DC analysis, we noticed that the SMG may play an important role in ARHL. Thus, the left and right SMGs were used as seeds in subsequent FC analysis to reveal their relationships with the whole brain. With the seed set at the left SMG, patients with ARHL showed lower FC with the right superior temporal gyrus (STG). In addition, the right SMG showed reduced FC with the right middle temporal gyrus (MTG) and increased connectivity with the left middle frontal gyrus (MFG) in patients with ARHL (Figure 2 and Table 2).

**Table 2:** Differences in DC and FC among patients with ARHL and healthy controls.

**Brain region**	**BA**	**Voxel size**	**Peak MNI coordinates (mm)**			**Peak *t* values**
			**X**	**Y**	**Z**	
**DC differences**						
Left supramarginal gyrus	48	32	−62	−30	27	−4.3595
Right supramarginal gyrus	48	33	58	−36	30	−4.3658
Left fusiform gyrus	20	43	−30	−25	−24	4.6355
Right parahippocampal gyrus	30	23	33	−17	−14	4.0522
**ROI: left supramarginal gyrus**						
Right superior temporal gyrus	42	35	63	−25	12	−4.1528
**ROI: right supramarginal gyrus**						
Right middle temporal gyrus	21	37	61	−31	12	−4.3556
Left middle frontal gyrus	40	26	−35	19	50	4.2344

The threshold was set at *p* < 0.05, family-wise error correction. BA, Brodmann area. MNI, Montreal neurological institute.

### 3.3. Correlation analysis

**Figure 3:**
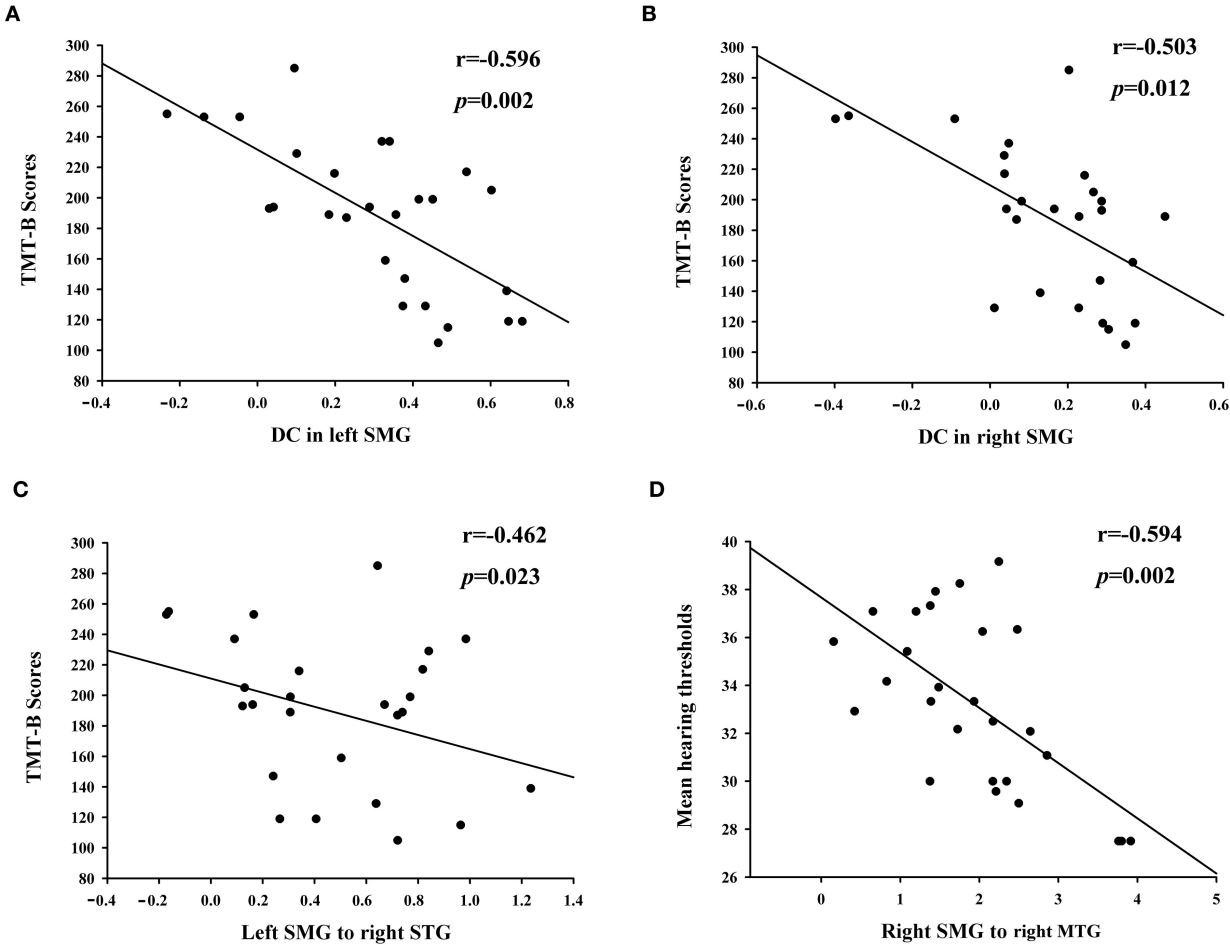
Correlation between altered brain functional network and clinical characteristics in patients with ARHL. The reduced DC in left and right SMG showed significant negative correlations with poorer TMT-B scores **(A,B)**. The TMT-B scores were negatively associated with left SMG connectivity to right STG **(C)**. The right SMG connectivity to right MTG was negatively correlated with the mean hearing thresholds **(D)**.

The reduced DC in the left and right SMGs showed significant negative correlations with poorer TMT-B scores (r = −0.596, *p* = 0.002; r = −0.503, *p* = 0.012, respectively) (Figures 3A,B). In the regions with altered FC of the left and right SMGs, the TMT-B scores were negatively associated with left SMG connectivity to the right STG (r = −0.462, *p* = 0.023), while the right SMG connectivity to the right MTG was negatively correlated with the mean hearing thresholds in patients with ARHL (r = −0.594, *p* = 0.002) (Figures 3C,D).

## 4. Discussion

To our knowledge, DC can quantify the overall connectivity of a node to all other nodes in the whole brain. Our DC findings proved that ARHL influenced not only the auditory center but also non-auditory brain regions. Consistent with previous research, subjects with ARHL performed worse on the TMT-B (17–19). They had lower DC values in the frontal gyrus and parietal lobe, as well as higher DC values in the cerebellum. In contrast, our patients with ARHL showed higher DC values in the FG and PHG. In terms of anatomy, FG is located close to the PHG and is known to decrease in size with increasing age in healthy people (20). The FG is related to high-order visual processing and word recognition. Chen et al. (17) confirmed that the effect of depression on the risk of dementia in aging was mediated by the hyper-synchronization of the hippocampus/FG, which supports our discovery in this study. In addition, we think that a high DC value of the FG might be associated with cross-modal reorganization after hearing deprivation. Long-term ARHL may lead to the enhancement of visual function or visual-auditory networks (21).

Furthermore, increased DC values have also been detected in the PHG, which is part of the limbic system. Kan et al. found high F-fluorodeoxyglucose (FDG) uptake in the PHG in ARHL patients with repetitive transcranial magnetic stimulation therapy (22). Traditionally, the PHG is a critical cognition-related structure that collects memories from the hippocampus and functions in learning and visuospatial tasks (23). In contrast to other studies, the activity of the PHG was not downregulated in patients with ARHL. Further study is required to explore the potential mechanism of the PHG.

Interestingly, we found that SMG was involved in ARHL compared with healthy controls. On the one hand, the SMG can be activated by pure tones and is causally implicated in auditory-motor integration (24). On the other hand, the SMG becomes a convergence zone of various networks associated with attention and verbal working memory (25). In ARHL patients with hearing loss (26) and bilateral sensorineural hearing loss (27), SMGs showed reduced connectivity with the cerebellum and nucleus accumbens, indicating that hearing deprivation had a negative impact on SMGs. An intracranial EEG study (28) provided evidence for SMG's role in encoding episodic memory, while some patients with ARHL in our study had poorer scores on the TMT-B. Thus, we conducted subsequent FC analysis using the left and right SMGs as two seeds since different sides of the SMG were reported to have diverse functions. Recent studies have implied that the left SMG is important for pitch memory processing, especially auditory memory retention (29). In the current study, the left SMG showed weakened connections with the temporal lobe in patients with ARHL. The right SMG is relevant to emotion recognition, which is known to change with age (30). In the present study, we did not find a significant difference in SAS and SDS scores, but patients with ARHL had relatively higher scores than healthy controls. Further study is required to explore the emotion of ARHL.

Several limitations need to be acknowledged in our study. First, our sample size was relatively small, and further research with a larger sample size is required to make the results more convincing. Second, we did not divide the ARHL subgroup according to different cognitive levels mainly due to the limited sample size, which will be taken into consideration in future studies. Furthermore, the participants cannot be completely isolated from the MR scanner noise that may influence the brain's functional network strength to varying degrees. This confounding factor should be taken into consideration in future studies. Finally, this is a cross-sectional study, and longitudinal work should be conducted to explore the progression of cognitive impairment.

## 5. Conclusion

Overall, this preliminary study mainly focuses on the difference in connections between ARHL and healthy controls, elaborating on the neural mechanism of cognitive deficits in ARHL. Our study might shift auditory diseases into central neural symptoms and contribute to early diagnosis. Moreover, this research could provide a potential therapeutic target for ARHL in the future.

## Data availability statement

The original contributions presented in the study are included in the article/supplementary material, further inquiries can be directed to the corresponding authors.

## Ethics statement

The studies involving human participants were reviewed and approved by research Ethics Committee of Nanjing Medical University. The patients/participants provided their written informed consent to participate in this study.

## Author contributions

SZ and JS drafted the manuscript for the work and acquired the clinical and fMRI data. WX helped to revise the manuscript critically for important intellectual content. WX and YX did the financial support, review, and final approval of the manuscript to be published. All authors have read and approved the final manuscript.

## References

[1] Gates GA Mills JH. Presbycusis. Lancet. (2005) 366:1111–20. 10.1016/S0140-6736(05)67423-5 16182900

[2] Ponticorvo S Manara R Cassandro E Canna A Scarpa A Troisi D. Cross-modal connectivity effects in age-related hearing loss. Neurobiol Aging. (2022) 111:1–13. 10.1016/j.neurobiolaging.2021.09.024 34915240

[3] Lin FR Metter EJ O'Brien RJ Resnick SM Zonderman AB Ferrucci L. Hearing loss and incident dementia. Arch Neurol. (2011) 68:214–220. 10.1001/archneurol.2010.362 21320988 PMC3277836

[4] Ouda L Profant O Syka J. Age-related changes in the central auditory system. Cell Tissue Res. (2015) 361:337–58. 10.1007/s00441-014-2107-2 25630878

[5] Qian ZJ Chang PD Moonis G Lalwani AK. A novel method of quantifying brain atrophy associated with age-related hearing loss. Neuroimage Clin. (2017) 16:205–9. 10.1016/j.nicl.2017.07.021 28808617 PMC5544491

[6] Ren F Ma W Li M Sun H Xin Q Zong W. Gray matter atrophy is associated with cognitive impairment in patients with presbycusis: a comprehensive morphometric study. Front Neurosci. (2018) 12:744. 10.3389/fnins.2018.00744 30405333 PMC6205975

[7] Ren F Ma W Zong W Li N Li X Li F. Brain frequency-specific changes in the spontaneous neural activity are associated with cognitive impairment in patients with presbycusis. Front Aging Neurosci. (2021) 13:649874. 10.3389/fnagi.2021.649874 34335224 PMC8316979

[8] Bidelman GM Price CN Shen D Arnott SR Alain C. Afferent-efferent connectivity between auditory brainstem and cortex accounts for poorer speech-in-noise comprehension in older adults. Hear Res. (2019) 382:107795. 10.1016/j.heares.2019.107795 31479953 PMC6778515

[9] Fitzhugh MC Hemesath A Schaefer SY Baxter LC Rogalsky C. Functional connectivity of heschl's gyrus associated with age-related hearing loss: a resting-state fMRI study. Front Psychol. (2019) 10:2485. 10.3389/fpsyg.2019.02485 31780994 PMC6856672

[10] Du Y Buchsbaum BR Grady CL Alain C. Increased activity in frontal motor cortex compensates impaired speech perception in older adults. Nat Commun. (2016) 7:12241. 10.1038/ncomms12241 27483187 PMC4974649

[11] Power JD Petersen SE. Control-related systems in the human brain. Curr Opin Neurobiol. (2013) 23:223–8. 10.1016/j.conb.2012.12.009 23347645 PMC3632325

[12] Xu Y Chen K Zhao Q Li F Guo Q. Short-term delayed recall of auditory verbal learning test provides equivalent value to long-term delayed recall in predicting MCI clinical outcomes: A longitudinal follow-up study. Appl Neuropsychol Adult. (2020) 27:73–81. 10.1080/23279095.2018.1481067 30470140

[13] Zuo XN Ehmke R Mennes M Imperati D Castellanos FX Sporns O. Network centrality in the human functional connectome. Cereb Cortex. (2012) 22:1862–75. 10.1093/cercor/bhr269 21968567

[14] Wang Y Zhong S Jia Y Sun Y Wang B Liu T. Disrupted resting-state functional connectivity in nonmedicated bipolar disorder. Radiology. (2016) 280:529–36. 10.1148/radiol.2016151641 26909649

[15] Xiong J Yu C Su T Ge QM Shi WQ Tang LY. Altered brain network centrality in patients with mild cognitive impairment: An fMRI study using a voxel-wise degree centrality approach. Aging (Albany NY). (2021) 13:15491–500. 10.18632/aging.203105 34106878 PMC8221306

[16] Buckner RL Sepulcre J Talukdar T Krienen FM Liu H Hedden T. Cortical hubs revealed by intrinsic functional connectivity: mapping, assessment of stability, and relation to Alzheimer's disease. J Neurosci. (2009) 29:1860–73. 10.1523/JNEUROSCI.5062-08.2009 19211893 PMC2750039

[17] Chen YC Yong W Xing C Feng Y Haidari NA Xu JJ. Directed functional connectivity of the hippocampus in patients with presbycusis. Brain Imaging Behav. (2020) 14:917–26. 10.1007/s11682-019-00162-z 31270776

[18] Xing C Chen YC Shang S Xu JJ Chen H Yin X. Abnormal Static and Dynamic Functional Network Connectivity in Patients With Presbycusis. Front Aging Neurosci. (2021) 13:774901. 10.3389/fnagi.2021.774901 35069176 PMC8766420

[19] Xing C Chen YC Tong Z Xu W Xu JJ Yin X. Aberrant brain functional hubs and causal connectivity in presbycusis. Brain Imaging Behav. (2021) 15:453–63. 10.1007/s11682-020-00386-4 32979169

[20] Shah M Kurth F Luders E. The impact of aging on the subregions of the fusiform gyrus in healthy older adults. J Neurosci Res. (2021) 99:263–70. 10.1002/jnr.24586 32147882 PMC8505033

[21] Rosemann S Thiel CM. Audio-visual speech processing in age-related hearing loss: Stronger integration and increased frontal lobe recruitment. Neuroimage. (2018) 175:425–37. 10.1016/j.neuroimage.2018.04.023 29655940

[22] Kan Y Wang W Zhang SX Ma H Wang ZC Yang JG. Neural metabolic activity in idiopathic tinnitus patients after repetitive transcranial magnetic stimulation. World J Clin Cases. (2019) 7:1582–90. 10.12998/wjcc.v7.i13.1582 31367617 PMC6658381

[23] Wang YK Shi XH Wang YY Zhang X Liu HY Wang XT. Evaluation of the age-related and gender-related differences in patients with primary insomnia by fractional amplitude of low-frequency fluctuation: A resting-state functional magnetic resonance imaging study. Medicine (Baltimore). (2020) 99:e18786. 10.1097/MD.0000000000018786 32011475 PMC7220210

[24] Li T Zhu X Wu X Gong Y Jones JA Liu P. Continuous theta burst stimulation over left and right supramarginal gyri demonstrates their involvement in auditory feedback control of vocal production. Cereb Cortex. (2022). 10.1093/cercor/bhac049 [Online ahead of print]. 35174862

[25] Yamasaki H Labar KS Mccarthy G. Dissociable prefrontal brain systems for attention and emotion. Proc Natl Acad Sci U S A. (2002) 99:11447–51. 10.1073/pnas.182176499 12177452 PMC123276

[26] Zhou GP Chen YC Li WW Wei HL Yu YS Zhou QQ. Aberrant functional and effective connectivity of the frontostriatal network in unilateral acute tinnitus patients with hearing loss. Brain Imaging Behav. (2022) 16:151–60. 10.1007/s11682-021-00486-9 34296381

[27] Xu XM Jiao Y Tang TY Zhang J Lu CQ Luan Y. Dissociation between Cerebellar and Cerebral Neural Activities in Humans with Long-Term Bilateral Sensorineural Hearing Loss. Neural Plast. (2019) 2019:8354849. 10.1155/2019/8354849 31049056 PMC6458952

[28] Rubinstein DY Camarillo-Rodriguez L Serruya MD Herweg NA Waldman ZJ Wanda PA. Contribution of left supramarginal and angular gyri to episodic memory encoding: An intracranial EEG study. Neuroimage. (2021) 225:117514. 10.1016/j.neuroimage.2020.117514 33137477

[29] Schaal NK Williamson VJ Kelly M Muggleton NG Pollok B Krause V. A causal involvement of the left supramarginal gyrus during the retention of musical pitches. Cortex. (2015) 64:310–7. 10.1016/j.cortex.2014.11.011 25577719

[30] Wada S Honma M Masaoka Y Yoshida M Koiwa N Sugiyama H. Volume of the right supramarginal gyrus is associated with a maintenance of emotion recognition ability. PLoS ONE. (2021) 16:e0254623. 10.1371/journal.pone.0254623 34293003 PMC8297759

